# Perception during use of force and the likelihood of firing upon an unarmed person

**DOI:** 10.1038/s41598-021-90918-9

**Published:** 2021-06-25

**Authors:** Adam T. Biggs, Joseph A. Hamilton, Andrew E. Jensen, Greg H. Huffman, Joel Suss, Timothy L. Dunn, Sarah Sherwood, Dale A. Hirsch, Jayson Rhoton, Karen R. Kelly, Rachel R. Markwald

**Affiliations:** 1 Naval Medical Research Unit Dayton, Dayton, OH USA; 2 Naval Special Warfare Command, Coronado, CA USA; 3 Naval Health Research Center, San Diego, CA USA; 4 Wichita State University, Wichita, KS USA

**Keywords:** Predictive markers, Stress and resilience

## Abstract

Stress can impact perception, especially during use-of-force. Research efforts can thus advance both theory and practice by examining how perception during use-of-force might drive behavior. The current study explored the relationship between perceptual judgments and performance during novel close-combat training. Analyses included perceptual judgments from close-combat assessments conducted pre-training and post-training that required realistic use-of-force decisions in addition to an artificially construed stress-inoculation event used as a training exercise. Participants demonstrated significant reductions in situational awareness while under direct fire, which correlated to increased physiological stress. The initial likelihood of firing upon an unarmed person predicted the perceptual shortcomings of later stress-inoculation training. Subsequently, likelihood of firing upon an unarmed person was reduced following the stress-inoculation training. These preliminary findings have several implications for low or zero-cost solutions that might help trainers identify individuals who are underprepared for field responsibilities.

## Introduction

Public safety depends on responsible use of force by military and law enforcement personnel, which necessitates a high degree of public trust. Unfortunately, 11% of deaths due to lethal force involve law enforcement firing upon an unarmed person^1^. When these errors occur, they not only affect the victim—they reverberate throughout the entire population. The potential implications have made exploring use-of-force issues a sprawling and multi-faceted endeavor that permeates aspects of human behavior, psychology, sociology, and law. For the individual, psychological ramifications are often revealed through the development of post-traumatic stress disorder (PTSD)^2–4^ with a specific emphasis on the change in symptoms before and after a lethal-force encounter^5^. Although psychological issues following armed conflict must be addressed, there is a significant advantage to understanding perceptual judgments during the event itself—when behavior will have its greatest consequences. The current study explored how stress during an armed conflict might impact perception of the event, whether training-related perceptual judgments were associated with independent use-of-force decisions, and how novel force-on-force training could limit the likelihood of firing upon an unarmed person.

Multiple initiatives have addressed stress and anxiety during use-of-force events, which is particularly critical as combat stressors may impose some of the largest performance impairments^6–9^. Research efforts tend to focus upon changes in general arousal^10,11^, the relationship between stress and memory^12–14^, or individual differences in reactions to anxiety^15–19^. Individuals do become inclined to shoot when they are anxious^20,21^, but practice alone is not sufficient to improve decisions in high anxiety circumstances^22^. It may be that anxiety increases stimulus-driven influences in attentional control while potentially impairing goal-directed behavior^23^. Unfortunately, these various findings describe the impact of stress *after* use-of-force or high-anxiety events; they do not address the perceptually-driven factors that might impact behavior *during* a use-of-force event. The latter is particularly important because issues affecting perception and judgment have the potential to change behavior prior to a use-of-force error rather than a post hoc evaluation of cause.

### Use-of-force training

For use-of-force training, the implications of previous research on stress are that: (1) stress will impair performance during an encounter that requires use of force; (2) training conditions may never be entirely realistic because many lethal use-of-force variables cannot be safely replicated during training; and (3) to the extent possible, these stressors must be replicated during training to prepare individuals for violent conflict. Therefore, the challenge is inducing realistic armed conflict stressors in a safe and reliable training environment to prepare individuals for the psychological and performance challenges of a firefight^24–26^.

One common tool for use-of-force training is non-lethal training ammunition (NLTA), which has been used in military and law enforcement training for decades^27,28^. Although NLTA rounds are not deadly, they can produce a significant pain sensation and leave temporary tissue damage if striking unprotected skin^29,30^. This ammunition provides several advantages as it can be fired from actual service weapons, can be fired safely upon living opponents, and provides an acute physical consequence of being shot. Compared to traditional firearms training tasks, such as firing upon static bullseye or silhouette targets with live ammunition, this method allows for more realistic force-on-force exercises during training. Done correctly, force-on-force training represents arguably the best and most underutilized method to prepare individuals psychologically for a use-of-force encounter^31^.

The underutilization of force-on-force training does not seem to be based on frequency or prevalence in training institutions. In fact, nearly every major law enforcement and military academy incorporates some type of force-on-force training^32^. However, there is little standardization, and its efficacy can be questioned due to a lack of accepted best practices and procedures. Essentially, the how-and-why this training matters has not been empirically documented. Recent efforts have started to explore this knowledge gap and demonstrate that NLTA in force-on-force exercises can induce significant physiological stress among participants^33,34^. This stress is due, at least in part, to the pain and wounds inflicted^29,30^ as well as the increased realism of minimally modified service weapons. For participants, this exposure is thought to inoculate them to the high stress conditions of an actual use-of-force encounter^35–39^. In theory, force-on-force training delivers a weakened version of the action and consequences of a true use-of-force encounter—much as a vaccine delivers a weakened version of a virus to prepare the immune system for the viral attack it will face.

Force-on-force methods also provide an interesting option to explore stress and perception while individuals are exchanging fire. It is difficult to quantify perception during an actual firefight, but force-on-force methods can allow for perceptual measurements during dynamic and safe use-of-force engagements. One possibility is to utilize embodied cognition methods to provide some insight into cognitive and/or perceptual differences due to actions and capabilities^40–46^. For example, research already suggests that holding a gun creates a bias to see guns in the hands of others^47^, and alters attentional priorities in scene viewing^48^. Furthermore, training with a weapon can alter information processing^49^, whereas anxiety can affect the functional relationship between physical distances and perceived threat^50^. The latter study suggests an underestimation of perceptual judgments in the presence of a threat. There is also evidence to support stress-induced impairments to perception and attention^51–53^. This combined evidence about embodied cognition suggests that collecting judgments of quantifiable elements during use-of-force situations might reveal individual differences in said judgments—regardless as to whether the effect occurs during perception or as a post-perceptual modification. The quantifiable elements may include variables such as counts of fired shots (i.e., *round counts*) and engagement duration.

### Current investigation

The current study explored perceptual judgments during force-on-force events within a novel close-combat training course. This course included a core stress-inoculation event pivotal to the novel course as well as close-combat assessments both pre-training and post-training. Given the ethical and practical challenges of altering a military training course for scientific inquiry, several independent but converging sources of evidence were identified across these various activities to explore the role of stress on perceptual judgments.

Key analyses involved the stress-inoculation event, where participants engaged a single peer in a controlled environment without obstacles. Both participants were armed with NLTA and given variable conditions across multiple engagements until the instructor deemed them to have completed the task—that is, successfully demonstrating mental resilience under fire. Dependent variables were measured following the stress-inoculation event to identify individual differences in situational awareness during the training exercise (cf. level 1 situational awareness through perception)^54,55^. Here, we conceptualize situational awareness as multiple perceptual judgments of varying accuracy that contribute to an overall understanding of the environment and activities occurring therein. Individual estimations were compared against physiological stress as measured by salivary cortisol before and after the stress-inoculation event. This physiological–perceptual evidence is one source to demonstrate the impact of stress on perceptual judgments.

For converging and more practical evidence, pre-training and post-training close-combat assessments required the individual to use NLTA while clearing a facility containing an armed hostile role player, an unarmed but aggressive role player, and an unarmed passive role player. These test events documented the likelihood of an individual firing upon an unarmed person in a realistic and independently conducted use-of-force assessment. The stress-inoculation event thus involved a training exercise designed to develop mental resilience, whereas the pre-training and post-training close-combat assessments involved more realistic use-of-force in a dynamic scenario. The term “force-on-force” could be applied to both activities. For clarity, the training exercise will be referred to as the stress-inoculation event and the pre-training/post-training assessments will be referred to as the close-combat assessments. Finally, the close-combat assessments documented whether force-on-force training could reduce the likelihood of an individual firing upon an unarmed person during an armed conflict.

### Hypotheses

We quantified situational awareness by identifying three objective sources of information about the stress-inoculation event, including temporal-awareness (time duration), threat-awareness (rounds fired), and pain-awareness (rounds hitting the participant). An overestimation would suggest a perceptual enhancement of the dangers similar to baseball players overestimating the size of a baseball based on their recent performance (cf. Witt and Proffitt 2005). Participants could be more likely to fire upon an unarmed person (e.g., during the pre-training and post-training close-combat assessments) because they overestimate the threat, according to this premise. Conversely, perceptual underestimation would suggest a loss of situational awareness with multiple explanations. Anxiety and elevated stress levels could increase the influence of stimulus-driven attentional control and reduce the efficacy of goal-directed behavior^23^, although attentional selection could explain underreporting of non-threat variables such as time elapsed when threat-related aspects receive attentional priority^56^.

Assuming a relationship between perception and stress, physiological biomarkers should correspond directly to measures of perception. To further characterize practical consequences, perceptual judgments during training should be related to use-of-force errors. This outcome would be represented statistically as a relationship between the stress-inoculation event and the close-combat assessments. Stress-related perceptual differences in the stress-inoculation event should predict the likelihood of firing upon an unarmed person during the close-combat assessment if the relationship is consistent and not the consequence of a training or measurement artifact.

## Results

### Stress validation and pain ratings

Participants engaged in the stress-inoculation event for an average of 27.92 min (*SD* = 9.43; Interquartile range = 8.62 s) with 4.87 min (*SD* = 2.63; Interquartile range = 2.38 s) spent actively firing upon one another. The remaining time included debriefs from the prior trial, new instructions, and receiving new ammunition. A mean of 99 rounds were fired at each participant (*SD* = 40.74) with 69 rounds (*SD* = 28.52) hitting them. Salivary cortisol levels were significantly lower prior to the event (*M* = 0.13 μg·dL^−1^, *SD* = 0.11) than immediately following the event (*M* = 0.83 μg·dL^−1^, *SD* = 0.29), (*Mean difference* = 0.71 μg·dL^−1^, *SD* = 0.27, 95% CI: 0.59 to 0.82), *t*(23) = 12.64, *p* < 0.001, *d* = 3.19. Averaged across the anticipated/hypothetical pain questions, participants reported a mean pain rating of 4.56 (*SD* = 1.77) on a 0–10 scale, which was significantly more than no pain, *t*(22) = 12.53, *p* < 0.001, *d* = 2.61. Taken together, non-lethal ammunition impacts were rated as causing “troublesome or uncomfortable pain”.

### Perception under direct fire

**Figure 1:**
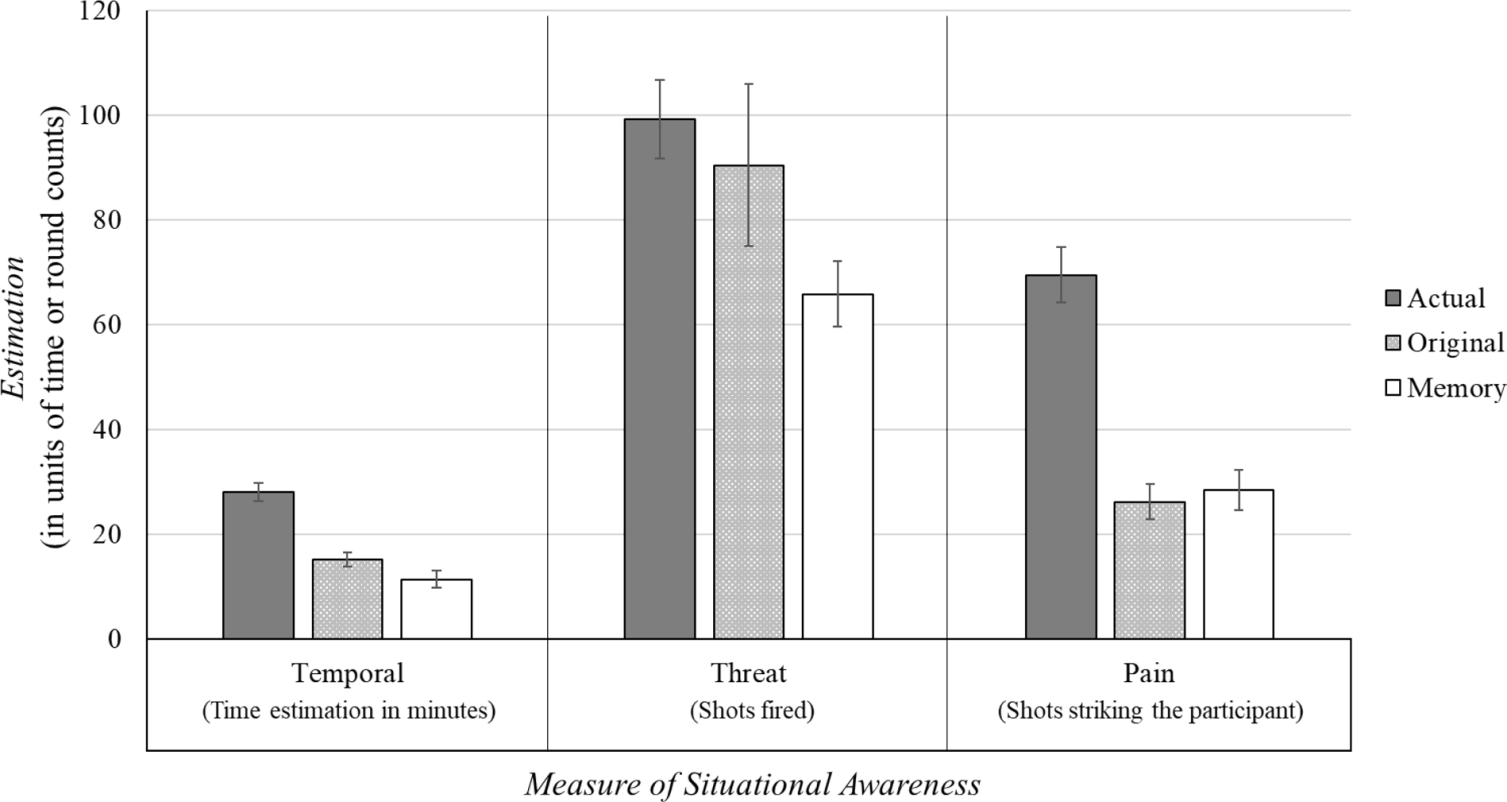
Reported values for the perceptual estimations in the stress-inoculation event, as separated by the actual event (dark grey bars, *actual*), reported values immediately after the event (light grey bars, *original*), and reported values a week after the event (white bars, *memory*). These reported measures are divided between estimations of situational awareness, including: temporal awareness (left, as measured by the total time duration of the event in minutes), threat awareness (middle, as measured by the number of shots fired by the participant at their opponent), and pain awareness (right, as measured by the number of rounds fired by the opponent that struck the participant). Pistol marksmanship and stress prior to the event were used as covariates in these measurements. Error bars show ± 1 standard error.

A repeated-measures MANCOVA was conducted to determine whether direct fire impacted perception for the different measures of situational awareness. This analysis included three dependent variables that measured a quantifiable aspect of situational awareness (*temporal,* measured as total time to complete the event; *threat*, measured as number of rounds fired during the event; and *pain,* measured as number of rounds hitting the participant) with assessments at three distinct time points (*actual*, or the actual event information; *original*, or estimates immediately following the event; and *memory*, or estimates made a week after the event). The two covariates were: (1) stress prior to the event, as measured by a salivary cortisol sample; and (2) pistol marksmanship, as measured by a live-fire pistol marksmanship assessment prior to the stress-inoculation event. This analysis used stress prior to the event rather than the change in cortisol because the intent is to address individual differences in stress upon entering the engagement. The change in cortisol could be affected by a participant’s prior experiences with NLTA; therefore, this would confound the results. Perceptual differences due to stress will be addressed in subsequent analyses. Multiple post hoc comparisons were corrected using the Bonferroni method. Data analyses were conducted using the IBM SPSS software (Version 27, IBM Corporation). See Fig. 1 for results.

There was a significant omnibus effect in the multivariate perception analysis indicating a change in situational awareness despite controlling for both stress prior to the event and marksmanship, Wilks’ *Λ* = 0.32, *F*(6, 80) = 10.19, *p* < 0.001, η_p_^2^ = 0.43. Univariate tests were examined for specific changes in situational awareness. All three variables indicated a significant change, including *temporal awareness* as measured through time estimations, *F*(2, 42) = 25.61, *p* < 0.001, η_p_^2^ = 0.55; *threat awareness,* as measured through estimated number of rounds fired, *F*(2, 42) = 3.32, *p* = 0.05, η_p_^2^ = 0.14; and *pain awareness*, as measured through estimations about number of rounds striking the participants, *F*(2, 42) = 20.28, *p* < 0.001, η_p_^2^ = 0.49. Post hoc comparisons were conducted on the specific estimations made for situational awareness.

For *temporal awareness*, the original reported duration of the event was significantly less than the actual duration, (Mean difference = 12.83 min, *SE* = 2.09, 95% CI: 7.38 to 18.27, *p* < 0.001, *d* = 1.55), and the memory report was significantly less than the original report (Mean difference = 3.83 min, *SE* = 1.09, 95% CI: 0.99 to 6.66, *p* = 0.006, *d* = 0.52). The actual amount and memory report were also significantly different (Mean difference = 16.65 min, *SE* = 2.01, 95% CI: 11.41 to 21.89, *p* < 0.001, *d* = 1.92). These results suggest that participants significantly underestimated temporal factors of the event itself, and this underestimation became even larger as the event consolidated into memory.

For *threat awareness*, the original reported number of rounds fired at the opponent was not significantly different from the actual amount, (Mean difference = 8.75 rounds, *SE* = 16.53, 95% CI: − 34.26 to 51.76, *p* > 0.95, *d* = 0.14), and the memory report was not significantly different from the original (Mean difference = 24.58 rounds, *SE* = 17.91, 95% CI: − 21.99 to 71.16, *p* = 0.55, *d* = 0.41). The significant univariate effect appears to be driven by a significant difference between the actual amount and the memory report, (Mean difference = 33.33 rounds, *SE* = 8.95, 95% CI: 10.06 to 56.60, *p* = 0.004, *d* = 0.94). These results suggest that participants significantly underestimated the threat once it consolidated into memory, although the changes between the event, immediate perception, and memory recollection were less robust than the temporal awareness changes.

For *pain awareness*, the original reported number of rounds striking the participant was significantly less than the actual amount, (Mean difference = 43.25 rounds, *SE* = 5.78, 95% CI: 28.23 to 58.27, *p* < 0.001, *d* = 1.88), but the memory report was not significantly different from the original report (Mean difference =  − 2.29 rounds, *SE* = 3.89, 95% CI: − 12.40 to 7.82, *p* > 0.95, *d* =  − 0.13). The actual amount and memory report were also significantly different (Mean difference = 40.96 rounds, *SE* = 7.02, 95% CI: 22.71 to 59.21, *p* < 0.001, *d* = 1.71). These results suggest that participants significantly underestimated consequences to them during the event, and this perception remained unchanged as it consolidated into memory.

For all three situational awareness variables (*temporal, threat, pain*), participants significantly underestimated the actual event based on the univariate analyses. The underestimation only differed on whether the change was significant following the initial event, as it consolidated into memory, or both. This consistent underestimation creates the potential for collinearity issues in further comparisons among the three measured components of situational awareness. Temporal awareness and pain awareness estimations were highly correlated for both the initial perceptual judgment (actual event − original estimation; *r*(22) = 0.52, *p* = 0.01) and the estimation as consolidated into memory (original estimation − estimation from memory; *r*(22) = 0.44, *p* = 0.03). Threat awareness did not significantly correlate with either temporal awareness or pain awareness for either the initial judgment or as consolidated into memory (all *p’*s > 0.33), but trended in the same direction as an underestimation. Because two of three estimations were highly correlated, and the third trended in the same direction, the variables were recoded into initially reported perceptual underestimations (*initial*) and perceptual underestimations as reported from memory (*memory*). For each of the three different measures, *z* scores were created to make them directly comparable, and then averaged together for a measure of relative perceptual underestimation among the participants. These initial and memory variables thus represent differences in situational awareness without collinearity issues among the individual component measures. The initial and memory variables were not significantly correlated, *r*(22) =  − 0.21, *p* = 0.33.

### Stress and perception

**Figure 2:**
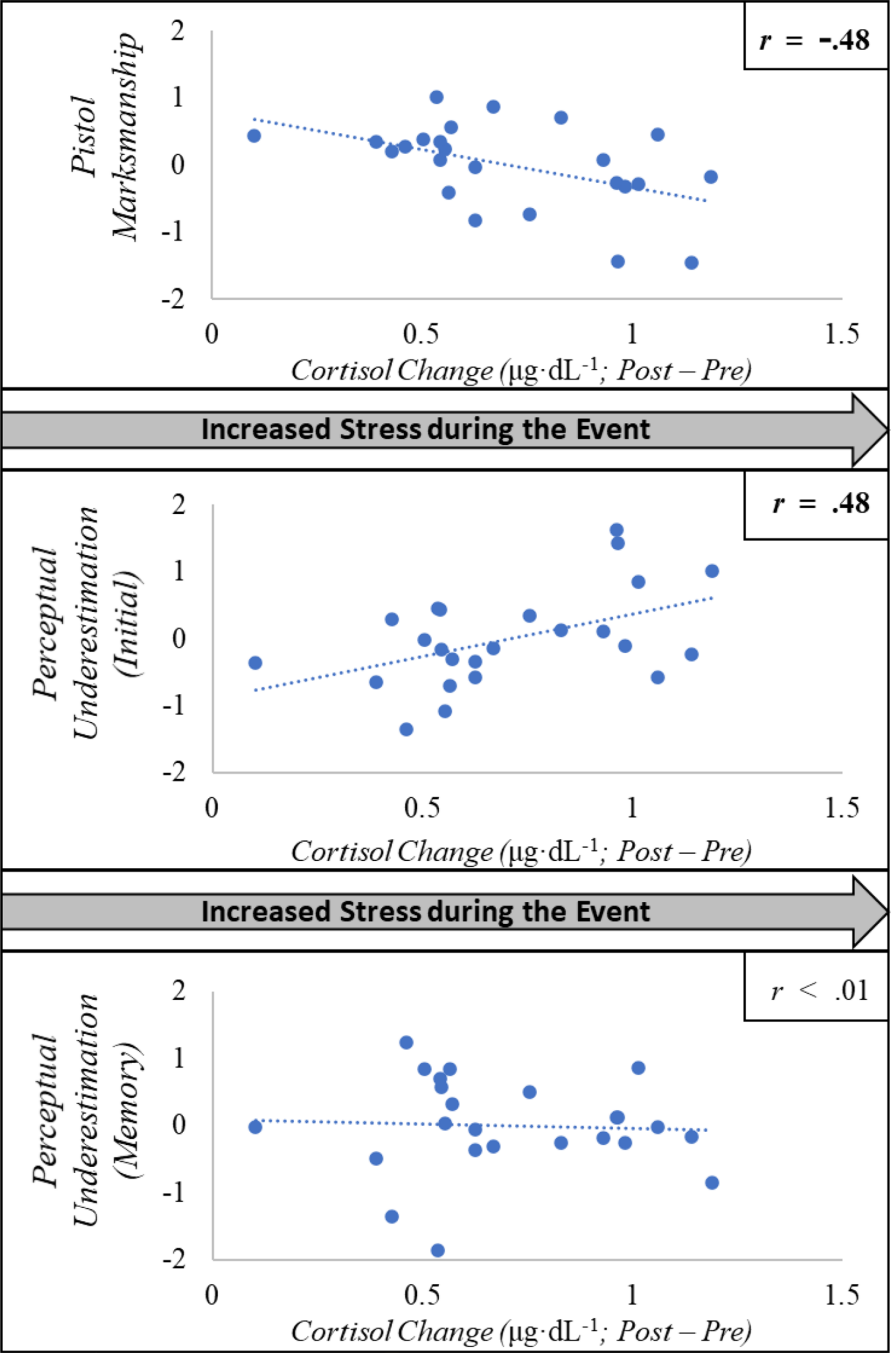
Scatterplots depicting the relationship between changes in stress level (as measured through salivary cortisol changes before versus after the stress-inoculation exercise) with average difference in *z* units for pistol marksmanship (top image), originally reported perceptual estimations across all three situational awareness variables (actual − original; middle image), and change as those estimations were consolidated into memory (original − memory; bottom image).

Physiological stress was calculated as the change in salivary cortisol from the measurement prior to the stress-inoculation event and immediately after the event. Perceptual underestimations were compared to physiological stress induced by the stress-inoculation event while controlling for pistol marksmanship through a hierarchical linear regression with physiological stress as the dependent variable. This hierarchical approach allows the variables to be assessed for their individual influence above and beyond the previous variables entered. The relationships between physiological stress and marksmanship, original report, and memory report are depicted in Fig. 2. The three predictor variables were entered in sequential steps based upon the order in which the tests were administered (pistol marksmanship, original perception reports, memory perception reports).

Marksmanship abilities did significantly predict the stress level of the training event (*R*^2^ = 0.23, *F*(1, 22) = 6.59, *p* = 0.02) with marksmanship as the sole predictor, β =  − 0.480, *t* = 2.57, *p* = 0.02. The force-on-force stress-inoculation training event was less stressful overall for more proficient marksmen. However, adding the original perceptual underestimations produced a significant change in the model (Δ *R*^2^ = 0.13, *F*(1, 21) = 4.28, *p* = 0.05), with both marksmanship as a significant predictor variable, *β* =  − 0.376, *t* = 2.07, *p* = 0.05, and perceptual underestimation as a significant predictor variable, *β* = 0.376, *t* = 2.07, *p* = 0.05. Whereas improved marksmanship skills reduced physiological stress during the event, perceptual underestimations were related to increased physiological stress—two largely independent effects that impacted physiological stress in divergent ways. Further underscoring the independence of the significant predictors, marksmanship and originally reported perceptual underestimations were not significantly correlated, *r*(22) =  − 0.28, *p* = 0.19. Adding the memory reports did not produce a significant change in the model (Δ *R*^2^ = 0.003, *F*(1, 20) = 0.10, *p* = 0.76).

### Likelihood of firing on an unarmed person

Although there was a relationship between physiological stress and a loss of situational awareness, the differences pertain solely to the stress-inoculation event. These perceptual differences cannot distinguish between individuals likely to make a use-of-force error—specifically, firing upon an unarmed person—because participants were always using force and not making threat/non-threat decisions. An additional assessment was made to determine if individual differences in perceptual judgments from the stress-inoculation event could be linked to firing upon an unarmed person in an independent close-combat assessment.

Prior to training, participants completed a close-combat assessment against role players to simulate a lethal-force encounter while using NLTA. During the assessments, participants faced three role players: (1) an armed and aggressive hostile; (2) an unarmed and aggressive non-hostile; and, (3) an unarmed and passive non-hostile. Note that all unarmed role players were instructed to comply with all commands given by the participant. This drill helped to assess performance before and after the novel close-combat training exercise. At the pre-training assessment, all 24 participants fired upon the armed hostile, 6 fired upon the unarmed and aggressive non-hostile, and 2 fired upon the unarmed and passive non-hostile. One individual fired upon both the aggressive and passive unarmed non-hostiles, and one individual fired upon only the unarmed and passive non-hostile. Thus, participants were divided into those who fired upon an unarmed role player (N = 7) and those who did not (N = 17) for subsequent analyses.

**Figure 3:**
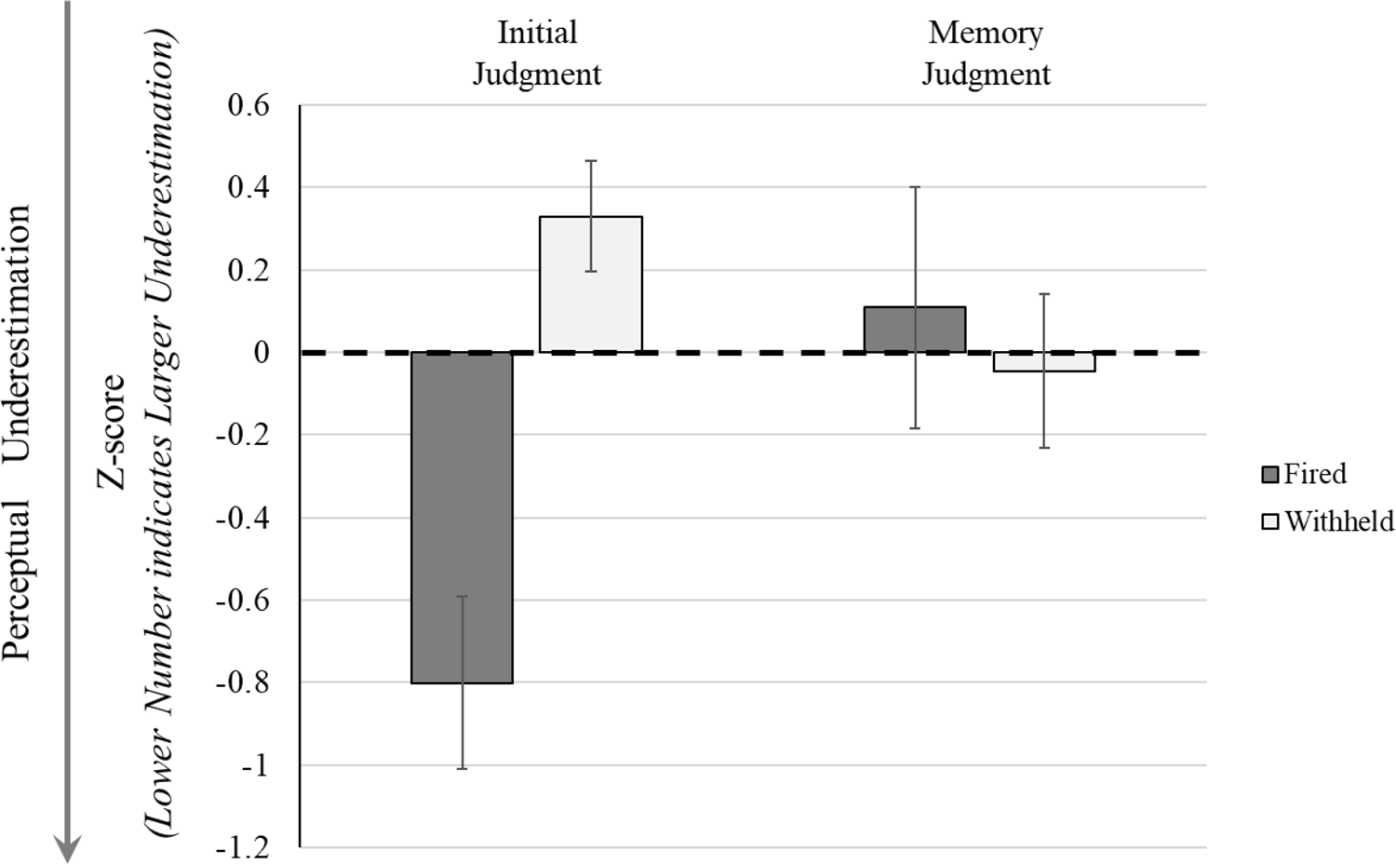
Perceptual differences from the stress-inoculation event between individuals who fired upon an unarmed person in the pre-training exercise and individuals who did not fire on an unarmed person (dark gray bars, *fired;* white bars, *withheld*). Left half of the figure indicates perceptual underestimations for the initial judgment, and the right half indicates perceptual underestimations for the memory judgment. Y-axis is the z-score combined situational awareness metric as averaged across the three components (temporal, threat, pain). Lower scores indicate a larger underestimation relative to the actual event. Error bars show ± 1 standard error.

A between-subjects MANCOVA was conducted to determine whether people who fired on an unarmed target during the force-on-force assessments also demonstrated perceptual underestimations during the independent stress-inoculation training event. This analysis included the between-subjects variable of whether the participant fired on an unarmed person during the pre-training assessment (*fired* or *withheld*) and the dependent variables of perceptual judgments from the stress-inoculation training event (*initial, memory*). Multiple post hoc comparisons were corrected using the Bonferroni method. Two covariates were used: (1) stress prior to the event, as measured by a salivary cortisol sample, and (2) marksmanship. As participants used a rifle during the force-on-force pre- and post-training assessments, the analysis used rifle (cf. pistol) marksmanship as the covariate. See Fig. 3.

There was a significant multivariate effect across the three different assessments interacting with whether the participant fired on an unarmed opponent, Wilks *Λ* = 0.50, *F*(2, 19) = 9.71, *p* = 0.001, η_p_^2^ = 0.51. There was a significant univariate effect for initial perceptual judgments, *F*(1, 20) = 5.91, *p* = 0.001, η_p_^2^ = 0.50. Participants who fired upon an unarmed person had significantly larger perceptual underestimations than those participants who withheld their shot and did not fire upon an unarmed person, (Mean *z*-score difference =  − 1.13, SE = 0.25, *p* < 0.001, 95% CI: − 1.66 to − 0.61). There was no significant difference for the memory perceptual judgments, *F*(1, 20) = 0.19, *p* = 0.67, η_p_^2^ = 0.01.

To better understand the likelihood of firing upon an unarmed person, a logistic regression was conducted to determine an odds ratio—how much more likely an individual would be to fire on an unarmed person based upon their perceptual underestimations during a training event. Only a single predictor (initial perceptual judgment) was used in the regression given the number of events in the data (7 instances of firing on an unarmed person) and the modified events per predictor rule of 5–9 events per predictor in a logistic regression^57^. Perceptual underestimations in the original reported values could accurately predict who fired upon an unarmed person through logistic regression, χ^2^(1) = 15.47, *p* < 0.001 (Cox & Snell *R*^*2*^ = 0.48, Nagelkerke *R*^*2*^ = 0.68) with 95.80% accuracy in classification and an odds ratio of 93.02 (95% CI: 1.66 to 5,211.65). For every full standard deviation below the group mean in perceptual estimations, the individual would be 93 times more likely to fire upon an unarmed person.

### Training-related changes

A final question involves whether force-on-force training would be effective in reducing the likelihood of firing on an unarmed individual. This assessment cannot be deemed causal given the lack of a randomized control group. However, observing the societal implications involved in use-of-force errors—particularly evident in recent years—it is prudent to identify a possible trend for future empirical exploration. During the post-training assessment, all 24 participants fired upon the aggressive hostile, 0 participants fired upon the aggressive non-hostile, and 0 participants fired upon the passive non-hostile. This raw comparison indicates that 29% of participants fired on an unarmed person at the pre-training event (7/24) and 0% fired upon an unarmed person at the post-training event (0/24). A 2 × 2 McNemar’s test was conducted on whether the individual fired upon an unarmed person or withheld the shot, and whether the event was pre-training or post-training. There was a significant reduction in the number of individuals firing upon an unarmed person from pre-training to post-training, χ^2^ (1) = 15.06, *p* < 0.001. This finding provides at least some evidence that novel force-on-force training with non-lethal training ammunition could help reduce these errors.

## General discussion

The current investigation provides insight into how stress influences perception during use-of-force and how combat stressors, most notably the impending threat of hostile fire, might impact the likelihood of firing upon an unarmed individual. Participants engaged in novel close-combat training designed to help individuals adapt to and overcome stress during a use-of-force situation. Following a stress-inoculation training exercise, participants significantly underestimated details associated with the actual event and demonstrated a lack of situational awareness. Self-reported perceptual underestimations correlated with increased physiological stress as measured by a change in salivary cortisol levels. Perceptual underestimations during the training exercise were related to the likelihood of firing upon an unarmed person in an independent close-combat assessment conducted prior to training. Finally, novel force-on-force training could reduce the likelihood of this critical use-of-force error, demonstrating the malleable—and more importantly, improvable—nature of performance under these circumstances.

Several mechanisms were hypothesized to explain the relationship between stress and perception during use of force^15^. Participants could have made errors because they overestimated threats, similar to how perceptual overestimations could relate to performance in a competitive environment^58^, but participants demonstrated perceptual underestimations rather than overestimations. Likewise, perceptual underestimations could be explained by selective attention if participants prioritized threat over other variables in the scenario^56^. This result should have been demonstrated by more accurate estimations for threat relative to other situational awareness variables. Although there is some minor supporting evidence, this hypothesis does not appear to be the primary influence. Instead, the best explanation would be stress- or anxiety-induced impairments to goal-directed behavior that boosted the relative influence of stimulus-driven attention^23^. For armed conflict, stimulus-driven behaviors would likely create a stronger bias to fire upon any stimulus presented because it could be a threat—ostensibly entering a simple see-something/shoot-something mindset once the weapon is drawn. This hypothesis appears to be the best explanation for three reasons: (1) participants did significantly underestimate multiple situational awareness variables, (2) these underestimations correlated with the physiological stress response, and (3) perceptual underestimations in a training event were related to use-of-force errors in an independent assessment.

An interesting finding is that the perceptual underestimations during the event were largely encoded and preserved in memory. The perceptual underestimation is supported by additional evidence, which supported perceptual underestimations when faced with a threat through systematic underestimations of distance to suspect^50^. However, stress generally impairs memory^17^, although there are techniques to help improve recollection^60,61^. The evidence here suggests a relatively clear memory with a trend toward underestimations specific to the threat. There are multiple possible explanations for this outcome. For example, the data was collected in a particularly intense training exercise. Discussion amongst participants after the event may have affected their recollection. This outcome could therefore be an artifact of the training environment, or lapses in memory could be attributable to poor encoding of the event itself rather than errors in retrieval. Additional evidence is required before making a firm conclusion either way.

The current evidence also contributes to a growing understanding about the psychological processes involved in use-of-force. The most thorough evidence of decision biases thus far has been documenting the role of prejudices and stereotypes in affecting bias during a threat response^61–66^. For use-of-force training implications, threatening contexts can produce more reliable learning effects than non-threatening contexts^67^ and inducing stress or emotion can actually aid identification in certain tasks^68–70^. Still, these findings are contrasted against straightforward and plausible outcomes such as how prior information influences the decision to shoot^71^, how individual posture might affect a lethal-force decision^72^, or how information accumulates throughout a lethal-force decision^73^. These latter findings highlight the need for empirical evidence to supplement subject matter expertise for training-related decisions. Subject matter expertise, although valid given the experience of the operators, lacks the rigor of empirical research as anecdotes dominate repeatable evidence.

These findings also highlight the role of cognitive functioning in threat assessments. For example, fear and anxiety can bias individuals to attend to threatening stimuli^56,74,75^. The implication is that cognitive processing might be particularly susceptible to manipulation during threatening scenarios. For example, an individual’s attention tends to become constrained to threat-related factors such as the weapon during a violent crime in lieu of broader details of the encounter itself^76,77^. These biases are especially noteworthy in the current context because attentional modification training may reduce this bias^78–80^, demonstrating the potential for training-related changes in cognition and behavior as they pertain to threat encounters. Moreover, the importance of cognitive failures emphasizes the need for alternative—or perhaps, complementary—methods to improve inhibitory control abilities through cognitive enhancement^81,82^, especially given the relationship between inhibitory control and the likelihood of making a lethal force decision error^83,84^. These complementary methods could include a wide variety of training programs, although methods such as biofeedback have demonstrated promising results^85–88^. Multiple alternative approaches demonstrate the multi-faceted nature of the problem and the need for similarly multi-faceted solutions.

### Limitations

Although the evidence does support these conclusions, it should be noted that this support remains preliminary. The overall underestimation in perceptual judgments does align with embodied cognition work demonstrating that changes in ability, affordances, or emotion can likewise influence perceptual interpretations within a given situation^89–94^. In armed conflict, individuals appear to lose situational awareness proportionate to their increase in stress levels that overload sensory processing capabilities. However, although the complexity and novel nature of this military training exercise demonstrates the potential for these improvements, a primary limitation remains the small sample size. Another limitation is the high variability in shooting performance within the stress-inoculation exercise. Additional studies may benefit from using a more standardized training event. Still, the results do produce an intriguing trend that warrants further empirical investigations of force-on-force training methods, although the direct correlation between perceptual judgments and physiological changes remains a highly novel finding.

### Summary

The most practical and important outcome is how this information could be used to bring about change in military and law enforcement training. These results could suggest a low-cost solution to identifying individuals who may not be ready for field operations by identifying people likely to fire on unarmed citizens before those trainees are certified ready for duty. Specifically, trainers could track quantifiable elements of training scenarios and see how well the trainee can maintain situational awareness. We provided three such assessments here through the situational awareness variables of time, threat, and pain, although these manipulations could be modified for further use. For example, environmental questions could extend to broader, scenario-based factors such as the size of the facility or number of rooms. Individuals who better maintain situational awareness appear less likely to fire on an unarmed person, and so as long as instructors have a method to quantifying aspects of the scenario, they could identify individuals with severe underestimations and loss of situational awareness who might be predisposed toward committing use-of-force errors. This potential future use requires additional scrutiny and refinement, but the potential advantage is a testament to how public safety and trust in our defense institutions benefits by combining the subject matter expertise of instructors with the rigorous empirical evaluations of scientific research.

## Method

### Ethical statement

Data collection was conducted in compliance with all applicable Federal regulations governing the protection of human subjects in the United States. The study protocol was approved by the Naval Medical Research Unit—Dayton Institutional Review Board under protocol number NAMRUD.2017.0010. All study participants completed research exercises on a voluntary basis and signed informed consent documents describing their rights as participants.

### Participants and data collection

Active duty military personnel (*N* = 24; 26.3 ± 0.3 years of age, 6.8 ± 0.2 years of service; 24 males) from the United States Army and United States Marine Corps participated in a novel close-combat training exercise. All data collection procedures were reviewed by the Naval Medical Research Unit Dayton Institutional Review Board under protocol NAMRUD.2017.0010 and all participants provided voluntary consent. To ensure voluntary compliance with the research efforts and to distinguish this data from the military training requirements, participants were informed of the elements specific to the training course and elements specific to the research data collection. Research efforts included the perceptual estimations, saliva samples to assess stress biomarkers, and pain estimations regarding the non-lethal training ammunition. Pre-training and post-training force-on-force assessments against role players were conducted as part of the military training exercise, and participants were informed during voluntary consent that they could deny including their data for any element of the research efforts. All participants provided voluntary consent for data inclusion consistent with these instructions. All participants completed the pre-training drill and the independent force-on-force training exercise. One participant could not complete the post-training drill due to illness unrelated to the training and research activities.

### Facility and equipment

Participants completed the pre-training and post-training assessments in a modular shoothouse manufactured by ‘Ultimate Training Munitions’ (UTM; Branchburg, NJ, USA; CAGE/NCAGE: KE396; DUNS: 519997550; GSA CONTRACT: GS-07F-5749P). The modular nature of its construction and design allows it to be used for multiple training iterations while adjusting the house layout to prevent the students from learning and predicting the facility. For the independent force-on-force training exercise, participants wore face protection, throat protection, and groin protection as required by the manufacturer recommendations. Participants wore typical military working uniform or utility uniform articles under the required personal protective equipment, including Service blouses, pants, and boots. For the stress-inoculation event, participants used either the Sig Sauer M18 or Glock 19 service pistol with UTM (https://utmworldwide.com) conversion kits and non-lethal training ammunition. For the pre-training and post-training room-clearing assessments, participants used their service-issued rifle (M27 or M4) with a modified bolt to allow them to fire non-lethal training ammunition also manufactured by UTM. Personal protective equipment included full combat gear to include plate carrier and armor plates as would be worn by the service member during an actual combat engagement (e.g., small arms protective inserts). Each service member setup their respective kit without input from the research team.

### Salivary biochemical sampling

To evaluate stress response to the stress-inoculation and pre-/post-training events, saliva samples were obtained acutely (immediately before and after) around each event and assessed for salivary cortisol. To reduce variability and sampling bias, participants were instructed to refrain from eating, drinking any beverages (less small amounts of water), smoking, vaping, or using smokeless tobacco within 30 min of testing and until all testing was completed. The stress-inoculation event took place after midnight for each participant; therefore, pre/post-event saliva samples were collected following a full day of wakefulness. Samples were obtained according to the manufacturer’s specifications (Salimetrics, LLC., Carlsbad, CA, USA), via SalivaBio Oral Swab technique. Immediately after collection, samples were placed on dry ice and subsequently frozen at − 80 °C until they were sent to Salimetrics, LLC. (Carlsbad, CA, USA) for analysis.

Analysis of the saliva was conducted via enzyme-linked immunosorbent assays (ELISA) for the quantification of cortisol. Samples were batch analyzed in duplicate. Immediately prior to performing the assay, samples were thawed to room temperatEaure, mixed, and then centrifuged for 15 min at approximately 3,000 RPM (1,500 × *g*). These steps have been shown to reduce mucins and viscosity of the samples to increase reliability and validity of testing.

### Pain survey

Immediately after completing the stress-inoculation event, participants rated the pain they experienced using a method for evaluating pain due to NLTA^29^. Although participants could not immediately rate the pain after being hit due to the logistical challenges of answering research questions while under direct fire, this survey allows each individual to rate the pain experienced by NLTA impacts after having recently experienced these strikes. Separate pain ratings were provided for all areas where the participant was hit with rounds above the waist, including hand, chest, and arms. Participants used an 11-point scale with the following anchors: 0 (*no pain at all*), 2 (*mild, annoying pain*), 4 (*uncomfortable or troublesome pain*), 6 (*distressing pain*), 8 (*intense pain*), and 10 (*worst pain imaginable*). One week after the stress-inoculation event, participants used the same scale to rate the pain experienced during the event for both an impact on the hand and an impact on the chest.

### Perceptual judgments

Following the methods developed by the embodied cognition literature, three measures were selected to represent various components of situational awareness during simulated armed combat: perception of the time, perception of the threat, and perception of pain. Participants were not aware they would be asked to report these variables after the event. Temporal awareness involved a time estimation for the overall event as time perception has been demonstrated to be fluid under stress^95–97^. This assessment was not directly tied to the threat itself, and as such, it represents an omnibus awareness of the overall scenario. Threat awareness was quantified using participants’ estimations of the number of rounds fired during the engagement. Finally, pain awareness was quantified using participants’ estimations of the number of rounds which hit them during the event. This element is unique to the individual shooter, quantifiable, yet still related to the threat manipulation. Each component—time, threat, and pain—was measured in three ways: actual, original, and memory. The actual duration or amount was recorded for the real event. Participants then made perceptual judgments immediately after the training exercise to document original perception, and consolidation into memory was measured by asking participants to estimate the same information a week later. Differences between measures provide the opportunity to document whether the actual event was misrepresented in perception, memory, or both.

### Marksmanship assessments

Because the tasks involved firearms and decision-making, marksmanship represents an orthogonal variable that could influence the outcome without necessarily influencing the decision^98^. Marksmanship was assessed through three drills using a pistol and rifle in close quarters. All participants completed all three drills for both pistol and rifle. All drills were performed at a distance of 7 m from paper targets, with live ammunition using Service-issued weapons; a CED7000 Shot-Activated Timer was used to provide an auditory “go” signal and to record shot times. The one-shot drill required the shooter to draw and fire a pistol from the holster or raise and fire a rifle from the low ready position. Dependent variables were speed to complete the shot and accuracy of the shot. The shot-to-shot reload drill required the shooter to fire one round, immediate reload a fresh magazine, and fire again upon the same target. Dependent variables were the time between shots and accuracy of both shots. The “bill drill” required the participant to draw and fire a pistol from the holster or raise and fire a rifle from the low ready position. Participants were instructed to fire 6 rounds into the target with speed (i.e., time to complete the drill) as the primary dependent variable. Marksmanship score was determined by creating a *z* score for each speed and accuracy metric with reverse-coded speed scores such that higher *z* scores indicate better performance. That is, a faster reaction time on a speed measure became a higher *z* score. The final marksmanship variable was calculated as a single composite score averaged across speed and accuracy *z* scores from the one-shot drill, the shot-to-shot drill, and the bill drill. Pistol and rifle *z* scores were calculated separately and used as covariates or predictor variables to identify individual differences in marksmanship skills based on the weapon used. The use-of-force assessment used a rifle as a primary weapon, whereas the stress-inoculation event used a pistol as the primary weapon.

### Single peer stress-inoculation event

**Figure 4:**
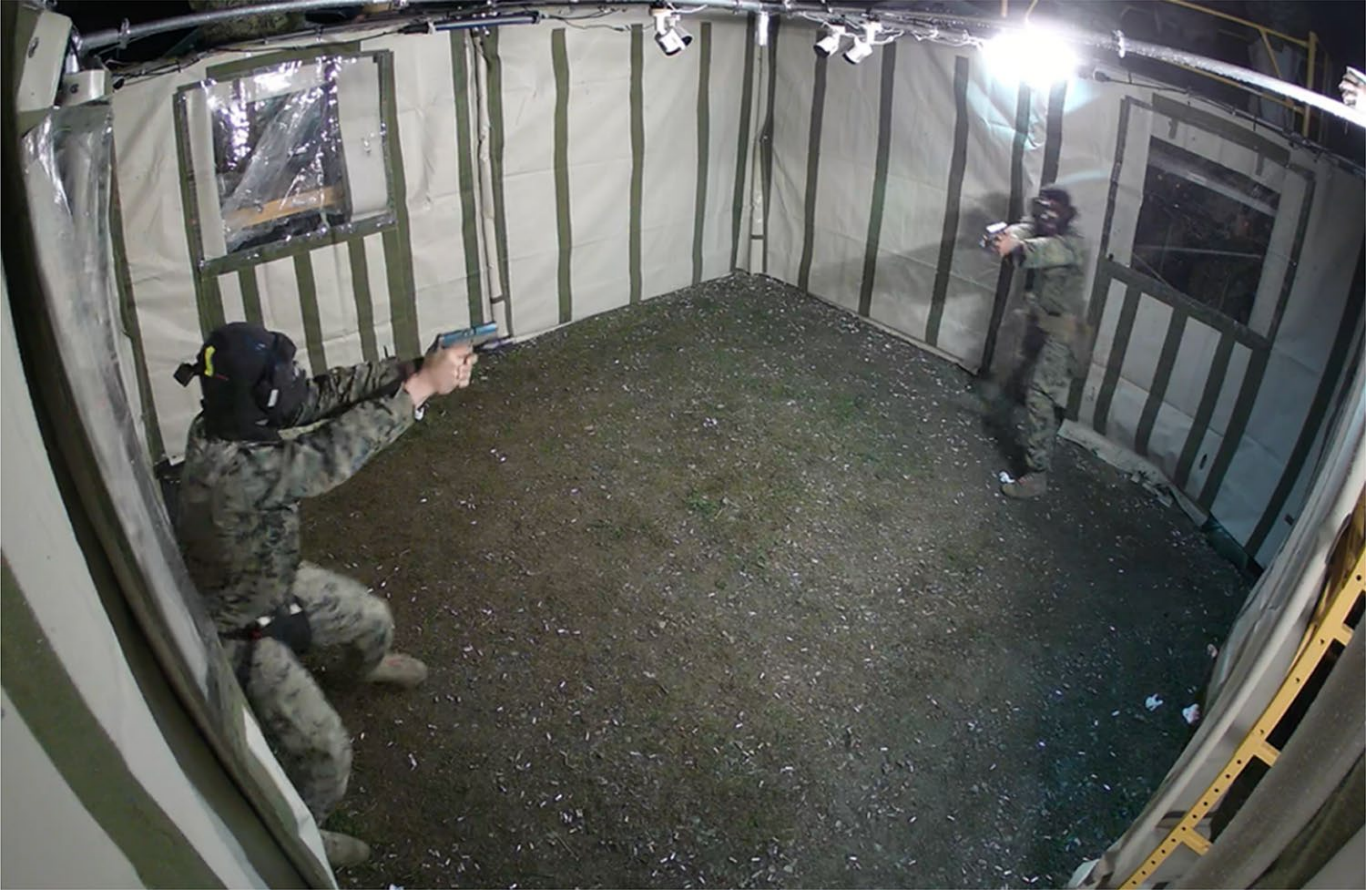
Sample image of the environment used during the force-on-force stress-inoculation training event against a single peer opponent. Participants were confined with a 4.9 m × 4.9 m space. Participants used pistols modified to fire non-lethal training ammunition and wore face, throat, and groin protection.

As a critical component of the close-combat training course, participants engaged in a force-on-force training exercise against a single peer opponent in a constrained space (see Fig. 4). The purpose of the exercise was to conduct weapons-handling drills under close fire conditions without cover, which allowed participants to experience, learn, and adapt to the challenges of conducting fundamental weapon manipulations while facing direct hostile fire. Prior to entering the shoothouse (4.9 m × 4.9 m), a saliva sample was collected from each participant as a means to assess stress biomarkers, specifically cortisol levels. Participants received individual directions as determined by the supervising instructor based upon individual training needs, such as particular goals or limitations relating to aim point, weapon handling, or other engagement variables as needed. For example, one participant may be working on deliberate accuracy or consistently using the front sight, whereas the other participant may be focused upon proper weapon manipulation procedure when reloading a magazine. Instructions could change each time participants received additional magazines of ammunition for the next trial within the training event. Upon the commence-fire signal, participants would begin the next drill in accordance with rules directed by the primary instructor until a cease-fire call was given or all parties ran out of ammunition. As such, all participants fired as many rounds themselves as were fired at them during the stress-inoculation event (although the number varied between different pairs of participants). All participants began the drill facing one another with a loaded, holstered pistol. Upon the start signal, participants were permitted to maneuver within the space however they chose, including moving closer or further away from their opponent but were not allowed to touch their opponent.

Event termination criteria were left to the primary supervising instructor to determine the point at which the individual servicemember had accomplished the training goals of performing rapid, repeatable, and safe weapon handling drills while under direct fire. Some participants completed drills additional times as required by the instructor. This instructor-determined criterion produced a different time duration of the event, number of rounds fired at each participant, and number of rounds hitting each participant. Researchers recorded the duration of the exercise and the number of rounds fired at each participant, with instructor-assisted determinations for the rounds hitting each participant. Once the instructor decided that the termination criteria had been met by the participant, the individual left the room and a second saliva sample was collected to determine cortisol change during the event. After providing the second saliva sample, participants walked to a separate tent for individual disclosure of pain-related information and to provide perceptual estimates. Additional estimations were provided one week later to compare initial perceptual estimations immediately following the event against recollection of the event as consolidated into memory.

### Force-on-force paradigm for pre-training and post-training assessment

**Figure 5:**
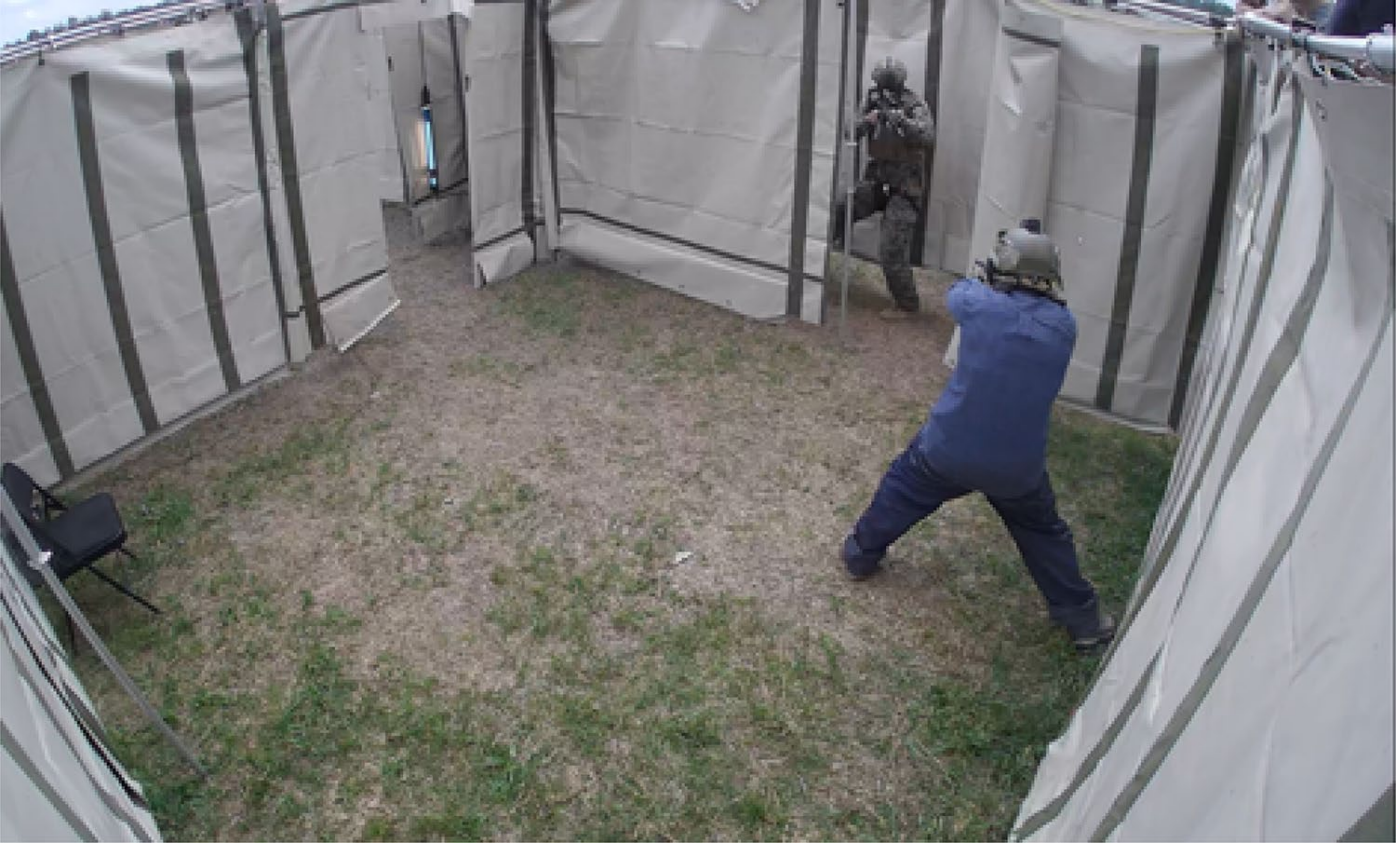
Sample image of the multi-room environment for the force-on-force training assessment. The participant (facing camera) searched the environment and encountered three role players. This image depicts the armed and aggressive hostile role player (back to camera).

Participants completed a realistic force-on-force exercise against role players as an assessment of training efficacy (see Fig. 5). During the pre-training and post-training assessments, participants were instructed to clear a modular structure while using non-lethal training ammunition. Participants received a mission brief about the simulation, but they were never provided information about the internal layout of the structure, number of role players in the structure, or the role players’ instructions. Inside the facility, three role players awaited participants: (1) an armed and aggressive hostile role player; (2) an unarmed and aggressive non-hostile role player; and, (3) an unarmed and passive non-hostile role player. The armed and hostile role player fired upon the participant with non-lethal training ammunition and received instructions from an independent observer watching the situation to determine when the hostile role player had been neutralized—at which point they fell to the ground and “died.” The unarmed and aggressive non-hostile role player moved toward the participant while shouting with both hands clearly empty. When approaching the participant, the aggressive non-hostile was instructed to begrudgingly comply with all commands as directed by the participant (e.g., freeze, drop to one knee, get down). The unarmed and passive non-hostile role player never approached the participant and immediately offered surrender or otherwise complied with all commands as given by the participant without attempting to offer threat or hostility of any kind. Primary dependent variables from these assessments were the number of rounds fired at unarmed participants, including the aggressive and passive unarmed non-hostiles.
